# Sustainability in the laboratory: evaluating the reusability of microtitre plates for PCR and fragment detection

**DOI:** 10.1098/rsos.242226

**Published:** 2025-05-21

**Authors:** Ane Liv Berthelsen, A. J. Paijmans, Jaume Forcada, Joseph Ivan Hoffman

**Affiliations:** ^1^ Department of Evolutionary Population Genetics, Faculty of Biology, Bielefeld University, Bielefeld, North-Rhein Westfalia, Germany; ^2^ Biological Sciences Division, British Antarctic Survey, Cambridge, UK; ^3^ Center for Biotechnology (CeBiTec), Faculty of Biology, Bielefeld University, Bielefeld, North-Rhein Westfalia, Germany; ^4^ Joint Institute for Individualisation in a Changing Environment (JICE), Münster University and Bielefeld University, Bielefeld, North-Rhein Westfalia, Germany

**Keywords:** sustainability, green lab, plastic crisis, reusability, microsatellite, genotyping error

## Abstract

Single-use plastics (SUPs) are indispensable in laboratory research, but their disposal contributes substantially to environmental pollution. Consequently, reusing common SUP items such as microtitre plates represents a promising strategy for improving laboratory sustainability. However, the key challenge lies in determining whether SUP reuse can be implemented without sacrificing data quality. To investigate this, we conducted a simple experiment to assess the impact of reusing microtitre plates on microsatellite genotyping accuracy. Plates previously used for polymerase chain reaction (PCR) and fragment detection were cleaned, opting for an environmentally friendly approach using regular soap, and then reused. Our results indicate that, while reusing PCR plates significantly increases genotyping error rates due to residual DNA contamination, detection plates can potentially be reused without compromising data quality. Our approach offers laboratories a practical and sustainable option for reducing SUP waste and costs while maintaining research integrity.

## 1. Introduction

Plastic waste has escalated into a global crisis. It is now highly improbable that any marine environments on Earth remain unaffected by plastic pollution [1]. Numerous policies and legislation aimed at managing plastic waste have been introduced at the national level (https://www.globalplasticlaws.org), and negotiations for a global plastics treaty are currently taking place [2]. However, sustainable laboratory management remains largely voluntary [3], and in biological, agricultural and medical research, plastic waste was estimated at around 5.5 million tonnes annually a decade ago [4]. This figure has undoubtedly risen since, despite the potential of sustainable laboratory management to reduce energy consumption, lower carbon footprints and cut the financial costs of running research facilities [5,6].

Fortunately, sustainable research practices have been gaining increasing attention. Each year, on the third Tuesday of September, researchers collect their plastic waste and post it on social media under the hashtag #LabWasteDay to raise awareness about the waste generated daily in research laboratories [7,8]. Concurrently, initiatives such as the Laboratory Efficiency Assessment Framework [9] and My Green Lab [10] provide researchers with tools to evaluate the environmental impacts of their laboratories and offer practical recommendations for improvements [11]. Often, these are simple adjustments such as reducing freezer temperatures from −80°C to −70°C, which can lower energy consumption by nearly a third [12]. Another example is the 6R Concept, introduced in 2023, which offers a framework for identifying sustainable solutions within existing protocols [13]. When applied to a neurobiology protocol, this framework achieved a 65% reduction in single-use plastic (SUP) waste [14].

SUPs have become a ubiquitous component of modern research laboratories globally. Their convenience, sterility and affordability have made them indispensable for a diverse array of experiments and protocols. However, SUPs are often classified as non-recyclable due to bio-safety concerns or the risk of contamination [11,15]. As a result, much of the plastic waste produced by research laboratories is either incinerated or sent to landfills [7,14]. While glassware can sometimes provide a practical alternative, it is not a feasible option for many items like microtitre plates or pipette tips. Consequently, reusing common SUP items offers a promising avenue for enhancing laboratory sustainability [7].

Microtitre plates, otherwise known as microplates or microwell plates, are flat plates containing multiple wells that allow large numbers of samples to be processed simultaneously in small volumes. They are used for a wide variety of applications in analytical research and clinical diagnostics, including immunoassays, colorimetric assays, tissue culturing and genetic screening [16]. The plastic polymers used in the manufacturing of microtitre plates vary depending on the specific application. For processes involving thermal cycling such as polymerase chain reaction (PCR), polypropylene is the most commonly used polymer because of its resistance to heat and chemicals. However, while these characteristics make polypropylene ideal for laboratory use, they also contribute to its persistence in aquatic environments, where it is one of the most frequently detected polymers in plastic pollution [17].

PCR is a crucial step in the amplification of microsatellites, which are codominant genetic markers used widely in both academic and industrial settings [18]. Microsatellite loci are known for their high mutation rates, which give rise to high levels of allelic diversity [18]. Amplification is achieved using oligonucleotide primers that bind to complementary sequences flanking the microsatellite. By incorporating fluorescent dyes into the PCR products, individual genotypes can be resolved through capillary electrophoresis and analysed using software packages such as GeneMarker (SoftGenetics, LLC, Pennsylvania, USA). This software implements the semi-automated calling of alleles by reference to marker panels containing information on expected allele sizes at each locus.

Unfortunately, even when sufficient amounts of high-quality DNA are available, microsatellites are susceptible to genotyping errors [19,20]. One of the primary sources of error is the mis-scoring of alleles, which can be exacerbated by the presence of stutter bands (artefactual peaks resulting from slippage during PCR amplification) and signal intensity differences between alleles [20]. To guard against genotyping errors, it is essential to manually review the automated allele calls produced by fragment analysis software such as GeneMarker. Additionally, it is advisable for all genotype calls to be cross-checked by at least one independent, experienced observer. Several approaches can be used to detect microsatellite genotyping errors, with the gold standard being the blind re-genotyping of a representative subset of samples [20].

Our laboratory routinely uses microsatellites for population genetic studies of wild animal populations [21–25]. As part of a long-term project on Antarctic fur seals, we have genotyped around 15 000 individuals sampled over three decades at between nine (≤2009) and 39 (2010–present) microsatellite loci [26–28]. Our standard microsatellite genotyping protocol (see Methods for details) processes batches of 96 samples in two consecutive steps—PCR amplification and fragment detection—each using a different type of microtitre plate. In the first step, the 39 microsatellites are PCR amplified in five separate multiplexes, each on its own ‘PCR plate’. The second step involves diluting the amplified products, mixing them with a size standard and transferring them to ‘detection plates’ for analysis on an automated capillary sequencer. This procedure requires a total of 10 microtitre plates for each batch of 96 samples.

**Figure 1.**
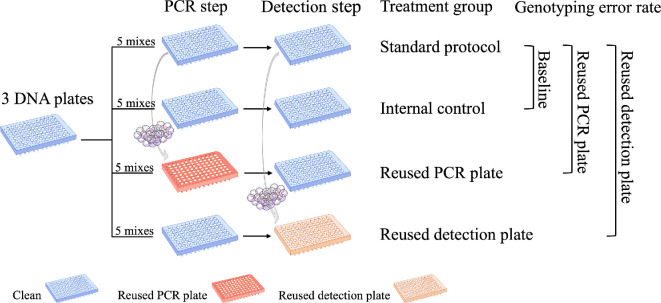
Overview of our experimental setup, which included four treatment groups. The ‘standard protocol’ treatment group used new PCR and detection plates and served as a reference to quantify genotyping error rates for the other treatment groups. The ‘internal control’ treatment group also used new PCR and detection plates and was used to estimate the baseline rate of genotyping error. The PCR plates and detection plates from the standard protocol treatment group were cleaned and reassigned to the ‘reused PCR plate’ and ‘reused detection plate’ treatment groups, respectively. A total of three plates of DNA samples (*n* = 288 samples) were genotyped at five multiplexes for each treatment group. The plates are colour-coded according to the legend. Original artwork by A.L.B.

To reduce SUP consumption in our laboratory, we performed a simple experiment to evaluate whether the PCR and/or detection plates could be reused without sacrificing data quality. We established four treatment groups within our experimental workflow: (i) ‘standard protocol’, (ii) ‘internal control’, (iii) ‘reused PCR plate’ and (iv) ‘reused detection plate’ (figure 1). The standard protocol, as previously described, used new PCR and detection plates (i.e. plates that had never been used before) and served as a reference for comparison with the other treatment groups. The internal control was identical to the standard protocol, also using new plates. By comparing the genotypes obtained from the standard protocol and the internal control, we could determine the baseline genotyping error rate.

As described below, we then cleaned the PCR and detection plates used in the standard protocol and reassigned them for use in the third and fourth treatment groups. In the third treatment group, reused PCR plates were used for the PCR step, while new detection plates were used for fragment detection. In the fourth treatment group, new PCR plates were used for the PCR step, while reused detection plates were used for fragment detection. By comparing the genotypes obtained from the standard protocol and the third treatment group, we evaluated the impact of reusing PCR plates on the genotyping error rate. By comparing the genotypes obtained from the standard protocol and the fourth treatment group, we evaluated the impact of reusing detection plates on genotyping accuracy.

We processed a total of 288 samples across three 96-well microtitre plates, following the experimental workflow outlined above. To evaluate the effects of reusing microtitre plates at different stages of the protocol, we implemented a formal Bayesian analysis of genotyping error rates. We hypothesized that (i) reusing microtitre plates should be feasible, in at least some circumstances, without significantly compromising data quality; however, (ii) the high sensitivity of PCR to trace amounts of DNA might introduce a risk of cross-contamination when reusing PCR plates, potentially increasing the genotyping error rate. Conversely, we anticipated that (iii) reusing detection plates would likely have a minimal impact on the genotyping error rate, as the capillary sequencer measures all signals, but only the strongest signals are scored.

## 2. Material and methods

### 2.1. Tissue sampling and DNA extraction

Tissue samples were collected from 288 Antarctic fur seals from an intensively studied breeding population at Bird Island, South Georgia (54°00024.800 S, 38°03004.100 W), during the austral summers of 2006−2007, 2015−2016 and 2020−2021. The seals were captured and restrained following protocols that have been established over more than 40 consecutive years of the long-term monitoring and survey program of the British Antarctic Survey. Pups were captured with a noosing pole on the day of birth and sampled from the umbilicus using piglet ear notching plyers. Each sample was stored individually in 20% dimethyl sulphoxide saturated with salt at −20°C. Total genomic DNA was later extracted using an adapted chloroform–isoamylalcohol protocol [29].

### 2.2. Standard microsatellite genotyping protocol

All samples were genotyped following our standard protocol, as detailed by Paijmans *et al*. [30]. In brief, 39 microsatellite loci were PCR amplified in five separate multiplex reactions using a Type It Kit (Qiagen). For this step, we used ultra-thin walled, non-skirted PCR plates (PCR trays; Rotilabo 96 well, standard, half frame, Roth Selection, Karlsruhe, Germany). Each plate contained 96 samples, including three positive controls to facilitate the standardization of microsatellite allele calling across plates. The PCR program included an initial denaturation step of 5 min at 94°C, followed by 28 cycles of 30 s at 94°C, 90 s at the annealing temperature (*T*_*a*_°C) specified for each multiplex reaction and 30 s at 72°C, with a final extension of 30 min at 60°C. The fluorescently labelled PCR products were transferred to hard-shell, fully-skirted detection plates (Fisherbrand™ 96-Well Semi-Skirted PCR Plates, Thermo Fisher Scientific, Waltham, MA, USA) before resolving them by electrophoresis on an ABI 3730xl capillary sequencer (Applied Biosystems, Thermo Fisher Scientific, Waltham, MA, USA). Allele sizes were automatically scored using GeneMarker v. 2.6.2 (SoftGenetics, LLC., State College, PA, USA), and the traces were manually inspected by two independent observers (A.L.B. and J.I.H., or A.J.P. and J.I.H.), with corrections being made where necessary to maximize genotype quality.

### 2.3. Experimental design

As outlined in the Introduction and illustrated in figure 1, our experiment included four treatment groups. The first (standard protocol) and the second (internal control) treatment groups followed the previously described workflow, both using new PCR and detection plates. We opted for a gentle, environmentally friendly approach to clean the plates using regular soap as described here: each plate was rinsed with distilled water and emptied 10 times before submerging it in soapy water for 2 h. After soaking, the plates were again rinsed and emptied 10 times before being left overnight on a paper towel to dry. The cleaned PCR and detection plates were subsequently reassigned to the third and fourth treatment groups, respectively. We retained information about the samples that were originally processed on each plate and ensured that no plate was reused for the same samples originally processed on it.

### 2.4. Evaluation of genotyping errors

Genotyping error rates were calculated based on discrepancies between the genotypes obtained from the standard protocol (reference treatment group) and those obtained from the three other treatment groups (internal control, reused PCR plate and reused detection plate). For each single-locus genotype, a binomial variable ‘mismatch’ (0 = match, 1 = mismatch) was computed. A ‘match’ indicates complete agreement between the genotypes from the standard protocol and the tested treatment group, while a ‘mismatch’ indicates a discrepancy at one or both alleles. Following Hoffman & Amos [20], we then calculated the error rate per reaction as the number of mismatching single-locus genotypes divided by the total number of genotypes compared.

### 2.5. Statistical analysis

We fitted a binomial Bayesian logistic mixed-effects model [31] to evaluate the effects of reusing PCR and detection plates on genotyping error rates. The response variable was ‘mismatch’, and the treatment group was included as a three-level fixed effect explanatory variable, with the internal control treatment group set as the reference (intercept) category. To account for heterogeneity arising from the use of different samples, DNA plates, multiplexes and loci, these variables were included as random effects in the model as follows:


$$log\left[\frac{p_{ijkl}}{1-p_{ijkl}}\right]=\alpha \left(t_{1}\right)+\beta_{1}\left(t_{2}\right)+\beta_{2}\left(t_{3}\right)+u_{i}+u_{j}+u_{k}+u_{l},$$

where *p*_*ijkl*_ represents the probability that the binary response variable mismatch is equal to 1 (mismatch observed) for an observation within the levels of the random effect variables sample ID (indexed by *i*), DNA plate (indexed by *j*), multiplexed reaction (indexed by *k*) and locus (indexed by *l*). The internal control treatment group was set as the reference category (*α*(*t*_1_)) against which the other treatment groups were compared. This analysis was performed using the *brms* package v. 2.20.4 [31], with three independent Markov chains being run for 100 000 iterations after a burn-in of 30 000 iterations with a thinning interval of 70. The model was fitted using a flat prior on the treatment groups and a Student *t-*test prior on the random effects (i.e. using the default *brms* priors). The trace plots were visually inspected, and model diagnostics such as R hat statistics and autocorrelation were generated using *brms* (accessible via the GitHub repository). All data analyses were implemented in R v. 4.2.1 [32] with RStudio v. 2023.09.1+494 [33].

## 3. Results

Multilocus genotypes were successfully generated for all four treatment groups for a total of 281 samples (97.6%). The corresponding genotyping error rates, calculated per reaction by reference to the standard protocol, are shown in table 1. The internal control and reused detection plate treatment groups exhibited similarly low genotyping error rates, at 0.005 and 0.004 per reaction, respectively. However, the genotyping error rate for the reused PCR plate treatment group was more than five times higher, at 0.028 per reaction.

**Table 1.** Per-reaction genotyping error rates for the internal control, reused detection plate and reused PCR plate treatment groups, calculated relative to the standard protocol treatment group. Due to variable amounts of missing data, the number of single-locus genotypes differs among the treatment groups. s.d., standard deviation.

treatment group	no. of single-locus genotypes	no. of mismatches	genotyping error rate ± (s.d.)
internal control	10 541	57	0.005 ± 0.004
reused detection plate	10 599	42	0.004 ± 0.002
reused PCR plate	9390	265	0.028 ± 0.011

**Figure 2.**
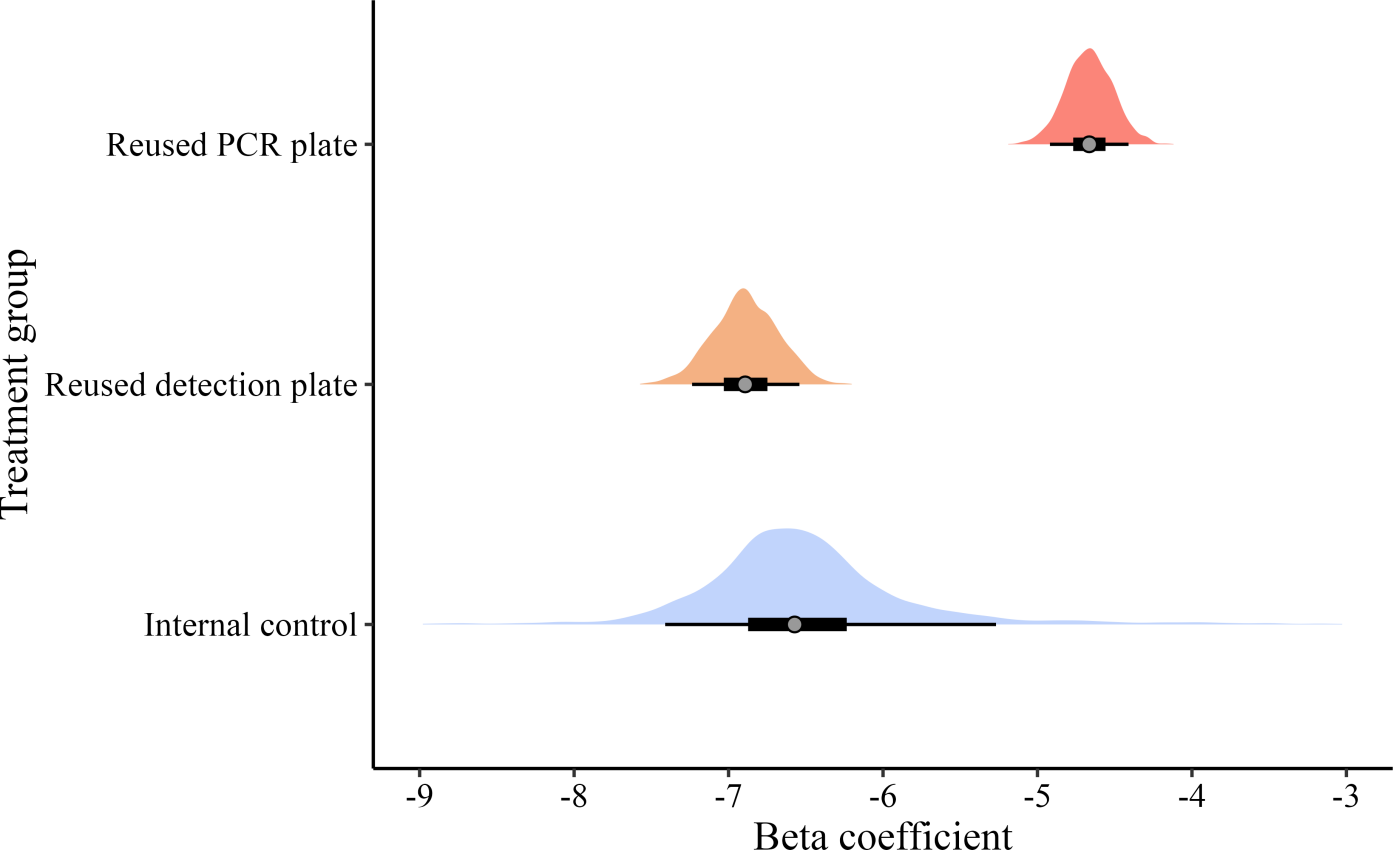
Posterior distributions of the beta coefficients (the degree of change in the response variable for every 1-unit change in the explanatory variable) of the internal control (blue), reused detection plate (orange) and reused PCR plate (red) treatment groups on genotyping errors. The grey points represent the mean posterior estimates, the thick black lines represent the 50% confidence intervals and the thin black lines represent the 90% confidence intervals.

To formally analyse variation in genotyping error rates among the treatment groups while accounting for heterogeneity among different samples, DNA plates, multiplexes and loci, we implemented a Bayesian logistic mixed effects model as described in the Methods. The model accounted for nearly 12% of the total variation in genotyping errors. The posterior distributions of the standardized beta coefficients of the genotyping error rates of the internal control and the reused detection plate treatment groups were similar, and their 95% confidence intervals (CIs) overlapped one another (figure 2; table 2). This indicates that reusing detection plates did not lead to a measurable increase in the genotyping error rate compared to the internal control. In contrast, the posterior distribution of the standardized beta coefficients of the genotyping error rate of the reused PCR treatment group was considerably higher than that of the internal control, with the 95% CIs of the two treatment groups not overlapping (figure 2; table 2). This indicates that reusing PCR plates resulted in a significantly higher genotyping error rate.

**Table 2.** Point estimates of beta coefficients and 95% confidence intervals (in square parentheses) from the Bayesian logistic mixed effect model testing for the effects of the three-level categorical fixed effect ‘treatment’ on genotype errors. Included are *R*^2^ (the proportion of the total variance explained by the model) and marginal *R*^2^ (the proportion of the total variance explained by the fixed effects alone) values.

	beta coefficient	[95% CIs]
fixed effects		
internal control	−6.572	[−7.658, −4.565]
reused detection plate	−0.320	[−0.739, 0.083]
reused PCR plate	1.907	[1.602, 2.209]
random effects		
sample ID	1.302	[1.100, 1.533]
DNA plate	0.388	[0.025, 2.864]
multiplexed reaction	0.348	[0.016, 1.571]
locus	1.273	[0.970, 1.759]
number of observations	30 835	
*R* ^2^	0.117	
marginal *R*^2^	0.001	

Beyond genotyping errors, we also noticed that the amount of missing data differed among the treatment groups. The internal control and the reused detection plate treatment groups had relatively few missing single-locus genotypes, at 3.0 and 2.3%, respectively. However, the reused PCR plate treatment group showed a much higher rate of missing data, at 13.4%. This suggests that reusing PCR plates not only increased genotyping error rates but also resulted in the loss of data. To investigate further, we revisited the original GeneMarker projects and classified the missing data into three categories: (i) failed PCRs, characterized by weak or absent PCR products; (ii) uninterpretable PCR products, characterized by high-intensity peaks that did not resemble microsatellite alleles; and (iii) probable contamination cases, where the genotypes could not be scored due to the presence of three or more apparent alleles. These categories accounted for 48.66, 24.47 and 26.87% of the failed reactions, respectively.

## 4. Discussion

We conducted an experiment to investigate the potential for reusing microtitre plates in our laboratory. We found that genotyping error rates for both the internal control and reused detection plate treatment groups were similar in magnitude to genotyping error rates observed in previous studies using microsatellites from our group, which range from 0.0013 to 0.0074 per reaction [20,25,34]. Importantly, these error rates fall well below the 1% threshold generally considered acceptable for microsatellite genotyping [35]. This indicates that detection plates can be reused in our fur seal genotyping protocol without compromising data quality. Nevertheless, despite this promising result, we recommend proceeding with caution. As we move forward with detection plate reuse in our laboratory, it will be important to maintain strict quality control by regularly monitoring genotyping error rates. This measure will help to ensure that any potential issues are promptly identified and addressed, safeguarding the integrity of our research.

The genotyping error rate for the reused PCR plate treatment group was more than five times higher than the background rate, exceeding the 1% threshold by nearly a factor of 3. This indicates that reusing PCR plates resulted in an unacceptable loss of data quality, supporting our original hypothesis that the high sensitivity of PCR to residual DNA may elevate genotyping error rates when reusing PCR plates. In contrast, the fragment detection step is less sensitive to residual DNA, as only the strongest signals are scored, and fluorescent signals are degraded by our cleaning protocol. However, our relatively gentle cleaning method appears to have been insufficient to fully eliminate DNA traces. Alternative approaches, such as treatment with DNase or 10% bleach, could be more effective. Bleach-based reagents are known to effectively degrade nucleic acids, but they can also be harmful to human health [36]. Other options include UV irradiation or autoclaving. However, these methods risk damaging the plates unless specifically designed materials such as autoclavable plates are used. Testing these alternatives would be worthwhile, although their effectiveness remains uncertain given that polypropylene is known to adsorb DNA [37]. This inherent limitation of polypropylene may ultimately constrain the effectiveness of cleaning methods, although using low-attachment plates made of polyallomer or low-binding polypropylene might help to mitigate this issue.

Our Bayesian mixed-effect model explained nearly 12% of the total variation in genotyping errors. As seen in previous research [20], much of this variation was attributable to the random effects of locus and sample ID. This is expected, as some loci are inherently more difficult to score, and DNA samples can vary in both the amount and quality of DNA available for PCR. Nevertheless, a substantial portion of the genotyping errors remains unexplained, suggesting that other, as yet unidentified, sources of genotyping error play a significant role. Possible contributors may include allelic dropout [38], false alleles resulting from PCR artefacts such as stutter bands [39], amplification errors due to low DNA quantity and/or quality [40] and scoring errors, including the misclassification of homozygotes and adjacent allele heterozygotes [20]. These complexities highlight the importance of implementing rigorous error-checking procedures, especially in studies relying on non-invasive sampling.

Unexpectedly, the reused PCR plate treatment group exhibited a higher percentage of missing data (13.4%) than both the internal control (3.0%) and the reused detection plate (2.3%) treatment groups. Manual inspection of the GeneMarker projects revealed that around half of the missing genotypes were due to failed PCR reactions, while the other half was roughly equally split between uninterpretable PCR products and cases of probable contamination. Although our use of a gentle cleaning protocol likely contributed to some of the missing data, the uneven distribution of missing genotypes among the treatment groups suggests that PCR amplification was generally less reliable on reused PCR plates. Consequently, our results suggest that reusing PCR plates can negatively affect both the quality and quantity of data obtained.

While cleaning PCR plates may pose challenges, reusing detection plates in our microsatellite genotyping protocol offers significant benefits in terms of reducing SUP consumption and costs. The extent of these savings will depend on how many times each detection plate can be reused, which is yet to be determined. However, even if each detection plate were reused just once, we would reduce SUP consumption by around 100 g and save around 33 EUR for each batch of 96 Antarctic fur seals genotyped. If the detection plates could be reused multiple times, these savings would be even greater. Currently, we estimate that our department uses around 500 detection plates per year. Reducing this number by 50% would save 5 kg of SUP and approximately 1650 EUR in consumables annually. Although these savings may seem modest at first glance, every step contributes to reducing our environmental footprint, and the cumulative benefits of implementing this simple measure will grow over time.

It is important to note that our findings are specific to our experimental protocol and cannot be directly generalized to other laboratories and contexts without further validation. However, given the widespread use of microtitre plates in analytical research, such as for qPCR and ELISA assays, it would be worthwhile to investigate whether our protocol could be adapted for these and other applications with appropriate modifications. We also acknowledge that reusing microtitre plates and other laboratory consumables may not always be appropriate, particularly in settings where precision is paramount, such as in medical diagnostics. Nevertheless, in less critical contexts, we see considerable promise in the reuse of microtitre plates. We hope that our findings can serve as a foundation for further research into the safe and effective reuse of laboratory materials across diverse research environments, and we encourage our colleagues to explore the potential for reusing SUPs in their own laboratories.

## 5. Conclusion

Our findings demonstrate that a common SUP item—microtitre plates—can be reused under certain conditions without any notable decline in data quality. This approach offers dual benefits: reducing plastic waste while also lowering costs, making it especially appealing for research groups or institutions operating on limited budgets. However, we emphasize the importance of continuous quality control to ensure consistently high data standards. More broadly, other SUPs in laboratory settings might also be suitable for reuse, offering further opportunities for waste reduction across various scientific fields.

## Data Availability

The script needed to reproduce the presented analysis, figure and tables has been provided as an R Quarto PDF file. It can additionally be accessed via GitHub [41]. The data are available via Zenodo [42]. Supplementary material is available online [43].
